# Case Report: Repeated Transcranial Magnetic Stimulation Improves Comorbid Binge Eating Disorder in Two Female Patients With Treatment-Resistant Bipolar Depression

**DOI:** 10.3389/fpsyt.2021.732066

**Published:** 2021-12-09

**Authors:** Domenico Sciortino, Giandomenico Schiena, Filippo Cantù, Eleonora Maggioni, Paolo Brambilla

**Affiliations:** ^1^Department of Pathophysiology and Transplantation, University of Milan, Milan, Italy; ^2^Department of Neurosciences and Mental Health, Fondazione Istituto di Ricovero e Cura a Carattere Scientifico (IRCCS) Ca' Granda Ospedale Maggiore Policlinico, Milan, Italy

**Keywords:** case report, binge eating disorder, intermittent theta burst stimulation, bipolar disorder, transcranial magnetic stimulation (TMS)

## Abstract

**Introduction:** Binge eating disorder (BED) is the most common eating disorder, affecting a large population worldwide. It is characterized by recurrent episodes of binge eating, with no compensatory behaviors. BED is often associated with psychiatric comorbidities, and still represents a challenge in terms of treatment strategies. In the last years, neuromodulation has represented a promising approach in the treatment of BED. We report the cases of two women, affected by Bipolar Disorder Type II (BD-II) and comorbid BED, whose BED symptoms improved after a course of accelerated intermittent Theta Burst Stimulation (iTBS).

**Methods:** We carried out a clinical study, involving neurostimulation on six patients with a treatment-resistant depressive episode. The trial consisted of a 3-week accelerated iTBS treatment, delivered to the left dorsolateral pre-frontal cortex. Clinical evaluation scales (Hamilton Rating Scale for Depression, Montgomery-Åsberg Depression Rating Scale, and Young Mania Rating Scale) were administered at baseline, after 2 weeks, and at the end of the stimulation cycle. Pharmacotherapy was maintained unchanged during iTBS treatment. Patients gave their informed consent both for the protocol and for the publication.

**Results:** The treatment was well-tolerated. Depressive symptoms only slightly improved; however, patients' binge episodes remitted completely, which was a serendipitous finding. BED symptomatology complete remission lasted up to 12 weeks follow-up.

**Discussion:** This is the first study regarding iTBS use in BED in comorbidity with BD-II. Further research is still needed to assess the efficacy of this technique in BED treatment.

## Introduction

Eating disorders (EDs) are serious psychiatric conditions characterized in varying degrees by abnormal eating, weight-control behaviors, and disordered body , which considerably impair physical health and disrupt psychosocial functioning (1–3). EDs show a high prevalence worldwide, with binge eating disorder (BED) being the most common ED: it affects ~3% of the adult population (4). BED is characterized by distressing, recurrent episodes of binge eating, with no compensatory behaviors (1). Patients show markers of cognitive and behavioral dyscontrol over eating habits, experiencing loss of control over the type and the amount of ingested food (5). Notably, psychiatric comorbidities are the norm in people with eating disorders (>70%), the most common of which being mood and anxiety disorders (5, 6), as exemplified in our case series.

The underlying mechanisms of BED are the subject of active research. The pathophysiology of these disorders is multifactorial, as it involves complex interactions between genetic and environmental factors (1). Regarding neurophysiologic mechanisms involved in BED, a widely accepted disease model suggests an altered balance between “bottom-up” drives and “top-down” cognitive control of eating behaviors (7, 8). In this framework, bottom-up drives are mainly related to ventromedial and limbic pathways, whereas top-down regulation appears to be mediated by dorsolateral circuitry, including the dorsolateral pre-frontal cortex (DLPFC) (8).

In this context, the DLPFC represents a crucial neural structure involved in self-regulation and inhibitory control (9). Reduced activity of the DLPFC might contribute to binge/purging behaviors (10). Indeed, the DLPFC seems to play a role in inhibiting food craving (11) and DLPFC hypometabolism has been observed in bulimic patients (10).

Thus, far, various therapeutic approaches have been proposed in BED treatment, including psychotherapy, pharmacological therapy, and neuromodulation techniques (1). Concerning neuromodulation, there is an expanding literature investigating this approach as a potential tool for the treatment of EDs (12). Neurostimulation strategies are capable of modulating cortical or subcortical excitability, producing therapeutic effects (12). Given the role played by DLPFC in eating behaviors, it has been hypothesized that modulating DLPFC activity might alleviate ED symptoms (10). Among the available neuromodulation techniques, repetitive transcranial magnetic stimulation (rTMS) represents a safe, non-invasive and well-tolerated option (13). rTMS generates a magnetic field that induces an electric current flow in the cerebral cortex below the coil. Various studies, including randomized controlled trials, have assessed the possible efficacy of rTMS in the binge spectrum disorders (12–15).

Diverse rTMS protocols are currently an object of study. Intermittent theta burst stimulation (iTBS) is an rTMS protocol in which pulses are applied in bursts of three, with an inter-burst interval of 200 ms (5 Hz, within the theta frequency range). Theta rhythms facilitate long-term potentiation (16), hence iTBS is thought to induce more rapid and longer-lasting effects on synaptic plasticity compared to conventional rTMS protocols (17). Moreover, iTBS seems to be more advantageous in terms of cost-utility (18). To our knowledge, no study about iTBS in BED has been published thus far, and only one study has investigated the effects of iTBS on EDs (19).

An additional strategy to improve the cost-utility of rTMS is to compress the administration, with multiple sessions of rTMS over a short period of time (accelerated rTMS). Recent studies have developed and tested accelerated iTBS protocols, in various psychiatric conditions (20–22).

In this article, we report the case of two women suffering from resistant bipolar disorder II (BD-II) depression and comorbid BED who experienced a complete remission of BED after receiving iTBS over the left DLPFC. iTBS was proposed in a clinical protocol framework, whose primary outcome was a reduction of depressive symptoms; the resulting improvement in BED symptomatology was a serendipitous finding. We proposed an accelerated iTBS protocol, which increases the feasibility of the procedure in terms of time expenditure, for both the patient and the operators. To our knowledge, no study has investigated accelerated iTBS in BED or other EDs before.

## Cases Description

### Patient 1

Patient 1 was a 45-year-old right-handed woman, who sought treatment for a depressive episode. The current episode onset was placed after a switch from sertraline to vortioxetine due to unbearable side effects (nausea and headache). She suffered from her first depressive episode at the age of 25, then alternating depressive and hypomanic episodes, which led clinicians to a diagnosis of BD-II.

The patient has also been suffering from EDs since her adolescence: she was diagnosed with anorexia nervosa at the age of 13; she then shifted to a bulimic eating pattern, with binge episodes followed by purging behaviors. This phase lasted for 10 years, followed by a complete remission until the age of 38. At this age she developed BED. Once a week or more she used to wake up at night and eat everything she could find in the fridge, including raw food. After these episodes she used to feel guilty and nauseated, but she did not show purging behavior anymore. She felt very uncomfortable due to either the loss of control or her weight gain. Her private psychiatrist then diagnosed her with BED, but apparently, she did not receive any psychological or pharmacological specific support. In the following years she had alternating periods of remission with periods of active disease. She denied alcohol or other psychoactive substances consumption. In the 2 months previous to our study, she had gained 12 kg, with 3–4 binge episodes per week on average. She denied medical comorbidities. When she first came to our attention, her therapy was the following: lamotrigine (150 mg daily), vortioxetine (20 mg daily). She was suffering from a depressive episode defined as mild according to the Hamilton Rating Scale for Depression (HAMD) and moderate according to the Montgomery-Åsberg Depression Rating Scale (MADRS) (Table 1).

**Table 1:** Clinical rating scales at baseline, after 2 weeks and after 3 weeks (end of the cycle).

	**Baseline**	**After 2 weeks**	**After 3 weeks**
			**(end of the cycle)**
Patient 1			
HAM-D	12	12	10
MADRS	25	23	23
YMRS	0	0	0
Patient 2			
HAM-D	22	18	17
MADRS	41	34	34
YMRS	0	0	0

*HAM-D, Hamilton Rating Scale for Depression; MADRS, Montgomery-Åsberg Depression Rating Scale; YMRS, Young Mania Rating Scale*.

### Patient 2

The second patient we are reporting is a 28-year-old right-handed woman, who came to our attention for a severe depressive episode. Her psychopathological onset is placed 10 years ago; she developed her first depressive episode with comorbid panic attacks. From that moment the patient has alternated phases of depression with sporadic episodes of elation, thus a sign of hypomanic episodes, which led clinicians to a diagnosis of BD-II. Her depressive phases used to have a seasonal pattern, with autumn or winter worsening. When she came to our attention (November 2020), the current episode had been lasting for 3 months, according to her seasonal pattern. She reported low consumption of alcohol in social circumstances and sporadic use of cannabis in her adolescence.

Regarding her ED, binging behaviors were reported to happen from the first diagnosis of depression, with various degrees of intensity and severity, and appeared to be more intense in depressive phases. Binge eating episodes during depressive phases used to be daily. No compensatory behaviors were ever observed. Notably, her first BED diagnosis was given in our center during her last depressive episode, as she had always been trying to hide her eating behaviors, even with physicians. Along with the current depression, she referred almost daily binge eating: after her dinner she used to go out and then buy and rapidly eat large amounts of high-fat food. This used to cause both physical and psychological distress.

In her past pharmacological history, many pharmacological therapies had been prescribed (i.e., valproate, fluoxetine, citalopram, venlafaxine, and bupropione) and when she came to our attention her therapy was clomipramine (150 mg daily) and pregabalin (225 mg daily). Her depressive symptoms at baseline were severe according to both MADRS and HAMD (Table 1).

## Methods

Our patients were recruited in an accelerated iTBS open label study, currently under review for publication. It included patients with treatment resistant depression, including both bipolar disorder and major depressive disorder, with the aim to explore the effects of accelerated iTBS in these conditions. Treatment resistant depressive episode in bipolar disorder was defined as the failure to respond to at least two separate monotherapeutic trials at an adequate dose for at least 8 weeks, or to monotherapy with one combination treatment (23). Previous neurostimulation treatments were an exclusion criterion. The original open label study included 6 patients, with none suffering from BED except for the two of this case report. The accelerated iTBS protocol consisted of 3 weeks TMS treatment delivered to the left DLPFC, with 3 days of stimulation in the first week, 2 days in the second week, and a single day in the last week, for a total of 6 days of stimulation. On each day of stimulation, each patient received 3 sessions of iTBS, with a 15-min pause between sessions. Hence, a total of 18 stimulations was performed. A single session of stimulation lasted 7 min. Thus, considering the pauses between sessions, the whole procedure lasted ~51 min each day. The site of stimulation was found in a parasagittal plane, 5 cm anterior to the site of MT determination. We delivered at 80% of the motor threshold (MT) triplet 30 Hz bursts, repeated at 5 Hz; 2 s on and 12.3 s off; 600 pulses per session. Our procedure was established according to an accelerated rTMS protocol which demonstrated to be as effective as the standard rTMS protocol in treating depressive symptoms (24). All the parameters were set according to International Safety Guidelines (25). TMS stimulation was delivered using a STM9000 Magnetic Stimulator (ATES Medica Device, Italy) with a 70-mm butterfly cooled coil. Our protocol was approved by the Local Ethics Committee, and patients were given an informed consent prior to their participation. Furthermore, the presented patients signed informed authorization for the publication of this case series in anonymous form. Clinical evaluation was carried out by administering the Hamilton Rating Scale for Depression (HAM-D), the Montgomery-Åsberg Depression Rating Scale (MADRS), and the Young Mania Rating Scale (YMRS). Clinical evaluation scales were administered at baseline, at the end of the second week, and at the end of the cycle of stimulation (3 weeks). Pharmacotherapy had been unvaried for at least 4 weeks prior to the enrollment and was maintained unchanged during the iTBS treatment. Notably, the primary outcome of our study was the reduction of depressive symptoms, evaluated with proper clinical scales. BED-specific clinical evaluation scales were not performed, as BED symptomatology was not an outcome of the original study. For the same reason, physical examination with body mass index measurement was not performed.

Notably, both patients received treatment between November and December 2019, before the Covid-19 outbreak.

## Results

**Figure 1:**
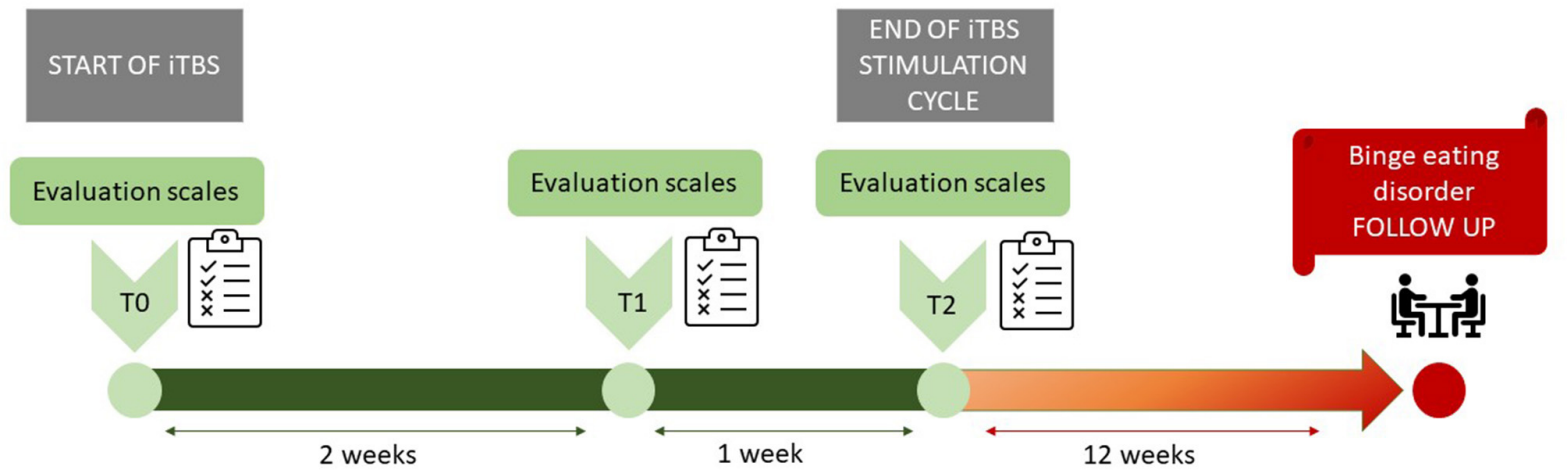
iTBS stimulation cycle and follow up. iTBS stimulation cycle lasted 3 weeks (in green). Evaluation scales were performed at baseline, after 2 weeks and at the end of the stimulation. Due to the serendipitous improvement in BED symptoms, a 12 weeks follow up was arbitrarily set (in red). The follow up consisted of a psychiatric interview.

The treatment was well-tolerated by both patients; no side effects were observed. Both patients concluded the treatment. Depressive symptoms only slightly improved, as shown in Table 1. More in detail, patient 1 kept showing a mild depression, as defined by both scales. Patient 2 improved from a severe depression into a moderate depression. However, the patients surprisingly reported the complete remission of their binge eating episodes. BED symptomatology improved with a remarkably similar pattern in both patients: it started to improve after the second week of treatment, with a reduction in the frequency of the episodes, then remitted completely after 2 weeks. Both patients spontaneously reported their BED improvement at the end of the iTBS cycle (3 weeks), during the last clinical evaluation of the study. As the two patients reported this unexpected outcome, we arbitrarily decided to set follow-up at 12 weeks (Figure 1). As previously mentioned, improvement in BED symptomatology was not an outcome of our study. Consequently, eating symptomatology was not assessed with appropriate evaluation scales. However, after 12 weeks follow-up, patient 1 reported a weight reduction of 4 kg. Patient 2 reported 2 kg weight loss. Both patients were very satisfied by the improvement in their BED, and described the received treatment as well-tolerated. Also, patient 1 asked for the feasibility of a further stimulation cycle in case of BED recrudescence. Patient 1 reported her disappointment with the lack of improvement in depressive symptoms.

## Discussion

To our knowledge, this is the first report regarding accelerated iTBS treatment in patients suffering from BED and comorbid BD-II depression. Our findings are consistent with the recent literature evaluating neuromodulation approaches as a therapeutic strategy for binge spectrum eating disorders (BED and bulimia nervosa). Indeed, recent reviews defined neuromodulation techniques as promising tools for these disorders (10, 13). In particular, concerning rTMS in BED, no open label or controlled study has been published thus far, but only a case report (26) which reported that high frequency rTMS delivered to the left DLPFC was able to improve BED symptomatology in a woman with refractory BED and comorbid depression. Moreover, the principal novelty of our case study is the use of an accelerated iTBS protocol in BED patients. The hypothetical therapeutic effect of enhancing DLPFC excitability in binge eating behavior is consistent with the role of DLPFC in inhibitory control. Stimulating DLPFC function could restore the putative imbalance between top-down and bottom-up regulation mechanisms of appetite proposed in EDs (7).

A major limitation of our case series is the lack of BED evaluation scales, such as the Binge Eating Scale (27), and the lack of proper physical evaluations. Once again, this is due to the serendipitous nature of our findings. Moreover, the open label nature of our report, in only two patients, does not allow us to draw any conclusion about the efficacy of accelerated iTBS in BED. Notwithstanding, such a dramatic improvement in BED after iTBS represents an intriguing result. Furthermore, as these results were not expected nor sought, it can be reasonably postulated that placebo effect was, if not absent, at least limited.

The clinical similarities between the two patients are a further matter of interest, and another point of strength of our report. The patients were both female, suffering from the same comorbid mood disorder. It is not clear if these similarities played a role in their improvement. Notably, the efficacy of rTMS protocols in BD is still undetermined (28, 29). In our cases, the improvement in BED seems to be independent from mood symptoms, which ameliorated poorly in both patients. This could suggest a specific action of iTBS on BED symptomatology.

## Conclusions

Neuromodulation use in EDs is currently a field of active research. In this framework, our cases study suggests that accelerated iTBS could be a potential therapeutic tool in BED. Though, rigorous randomized controlled trials with proper evaluation scales will be required to evaluate the efficacy of accelerated iTBS treatment in BED and confirm our findings.

## Data Availability Statement

The original contributions presented in the study are included in the article, further inquiries can be directed to the corresponding author.

## Ethics Statement

The studies involving human participants were reviewed and approved by Local Ethic Committee. The patients/participants provided their written informed consent to participate in this study. Written informed consent was obtained from the individual(s) for the publication of any potentially identifiable images or data included in this article.

## Author Contributions

DS wrote the first draft of the manuscript, along with GS, PB, and FC. EM revised the manuscript. All authors agreed with the final content of the manuscript.

## Funding

EM was supported by the Italian Ministry of Health (GR-2018-12367789).

## Conflict of Interest

The authors declare that the research was conducted in the absence of any commercial or financial relationships that could be construed as a potential conflict of interest.

## Publisher's Note

All claims expressed in this article are solely those of the authors and do not necessarily represent those of their affiliated organizations, or those of the publisher, the editors and the reviewers. Any product that may be evaluated in this article, or claim that may be made by its manufacturer, is not guaranteed or endorsed by the publisher.
